# Phytotherapeuthics Affecting the IL-1/IL-17/G-CSF Axis: A Complementary Treatment Option for Hidradenitis Suppurativa?

**DOI:** 10.3390/ijms23169057

**Published:** 2022-08-13

**Authors:** Katrin Witte, Robert Sabat, Ellen Witte-Händel, Kamran Ghoreschi, Kerstin Wolk

**Affiliations:** 1 Psoriasis Research and Treatment Center, Charité—Universitätsmedizin Berlin, 10117 Berlin, Germany; 2 Interdisciplinary Group of Molecular Immunopathology, Dermatology/Medical Immunology, Charité—Universitätsmedizin Berlin, 10117 Berlin, Germany; 3 Inflammation and Regeneration of Skin, BIH Center for Regenerative Therapies, Charité—Universitätsmedizin Berlin, 13353 Berlin, Germany; 4 Department of Dermatology, Venereology and Allergology, Charité—Universitätsmedizin Berlin, 10117 Berlin, Germany

**Keywords:** acne inversa, IL-1, IL-17, G-CSF, TNF-α, LCN2, neutrophilic granulocytes, polyphenol

## Abstract

Hidradenitis suppurativa (HS; also designated as acne inversa) is a chronic inflammatory disease characterized by painful skin lesions that occur in the axillary, inguinal, gluteal and perianal areas of the body. These lesions contain recurring deep-seated, inflamed nodules and pus-discharging abscesses and fistulas. Affecting about 1% of the population, this common disease has gained appropriate clinical attention in the last years. Associated with numerous comorbidities including metabolic syndrome, HS is considered a systemic disease that severely impairs the quality of life and shortens life expectancy. Therapeutic options for HS are limited, comprising long-term antibiotic treatment, the surgical removal of affected skin areas, and neutralization of TNF-α, the only approved systemic treatment. Novel treatment options are needed to close the therapeutic gap. HS pathogenesis is increasingly better understood. In fact, neutrophilic granulocytes (neutrophils) seem to be decisive for the development of the purulent destructive skin inflammation in HS. Recent findings suggest a key role of the immune mediators IL-1β, IL-17A and G-CSF in the migration into and activation of neutrophils in the skin. Although phytomedical drugs display potent immunoregulatory properties and have been suggested as complementary therapy in several chronic disorders, their application in HS has not been considered so far. In this review, we describe the IL-1/IL-17/G-CSF axis and evaluate it as potential target for an integrated phytomedical treatment of HS.

## 1. Hidradenitis Suppurativa—A Debilitating Disease with High Medical Need

Hidradenitis suppurativa (HS; also designated as acne inversa) is a chronic inflammatory skin disease characterized by the recurrent appearance of painful inflamed nodules, and pus-discharging abscesses and tunnels in the intertriginous skin areas [1]. The axillary, inguinal, gluteal and perianal sites are most commonly affected. Usually starting in early adulthood, HS affects both sexes with an overall similar frequency [2,3]. The worldwide prevalence of HS is estimated at about 1% [1]. HS is associated with numerous comorbidities including metabolic syndrome, spondyloarthritis, inflammatory bowel disease as well as non-alcoholic fatty liver disease (NAFLD), and is therefore seen as a systemic disease [4,5,6,7,8,9,10,11]. Importantly, the number of concomitant diseases correlates with the duration from manifestation of first symptoms until HS diagnosis [12]. In Germany, this delay in diagnosis of HS was shown to be ~10 years on average [12].

A closer look at the disease reveals the huge burden HS patients have to carry. With the painful skin lesions secreting malodorous pus in intimate body sites, HS has a large negative impact on the quality of life of those patients [13,14]. In fact, discomfort, an impaired sexual health and body image, anxiety, depression, stigmatization, social exclusion, and passive forms of indirect self-destructiveness are frequently associated with HS [15,16,17,18,19].

From the etiological point of view, genetic factors play a role in about 33% of patients as suggested from a positive family history for HS. In the majority of these patients, the contributing genetic factors are unknown; only in a small proportion of patients they have been elucidated and involve molecules of the Notch signaling pathway [1,20,21]. Moreover, life style factors, such as obesity (due to immunological shift, mechanical friction of skin folds, wetness and microbiome alterations) and in particular smoking habits are suggested to trigger the disease [20,22]. These factors are supposed to induce subclinical inflammation around hair follicles and infundibular hyperkeratosis and acanthosis, leading to follicular plugging, dilatation and rupture of the pilo-sebaceous unit [20]. As a consequence, local immune cells get activated by released components of the pilo-sebaceous microbiome and damaged host cells. Inflammatory cytokines secreted by immune cells then activate tissue cells to drive immune cell infiltration from peripheral blood into the skin [20]. Resulting chronic inflammatory processes lead to destruction of normal skin architecture, with formation of abscesses, fistulas and scarring. Especially neutrophilic granulocytes, by secreting matrix-degrading enzymes and reactive oxygen species, seem to play a role in tissue destruction [23,24,25]. G-CSF, the main regulator of neutrophilic granulocyte survival, was recently shown to be a key cytokine in HS pathogenesis and potential therapeutic target [24].

Despite the high prevalence of HS and severe physical and mental suffering of the patients, the therapeutic options for this disease are still limited [1,26,27]. This is in contrast to other common chronic inflammatory skin diseases, such as psoriasis, for which we have numerous very effective innovative drugs [28]. In fact, HS therapy relies primarily on long-term antibiotic treatment and the surgical excision of affected skin areas. Importantly, these interventions are not associated with long-lasting reduction of the impairment of patients’ quality of life [13]. Furthermore, trapped hair fragments are frequently found in lesioned HS skin, resulting in suggestion of laser epilation as complementary treatment [29]. Moreover, the TNF-α-neutralizing antibody adalimumab is currently the only approved systemic therapy for HS. Since only a part of the patients respond to anti-TNF-α therapy with relevant symptom reduction, there is still an urgent need for novel therapy options [30]. Contraindications to, refusal of certain therapy elements, or adverse effects such as *Clostridium difficile* infection in context of long-term antibiotic treatment are also relevant factors underlining this need [31].

For several chronic inflammatory diseases, conventional medicine can be complemented by phytomedical drug-based therapy options. For some of them, such as Colitis ulcerosa, Morbus Crohn and early rheumatoid arthritis, phytotherapy is even part of respective S3 guidelines [32]. In contrast, phytomedicals are not considered for complementary HS therapy so far although there is a positive perception of alternative complementary treatment concepts by patients and dermatologists [33,34]. In this review, we describe cytokines that regulate the migration, persistence and activation of neutrophilic granulocytes (neutrophils) in the skin of HS patients and evaluate the potential to influence them by phytomedical drugs.

## 2. Regulation of Neutrophilic Granulocyte Homeostasis

Neutrophilic granulocytes are the most abundant cell type among leukocytes. They are part of the first-line innate host defense against tissue-invading microbes. These short-lived cells exert their function through reactive oxygen production, the release of molecules from intracellular vesicles (enzymes, proteases, antimicrobial peptides, chemokines, cytokines), phagocytosis, and neutrophil extracellular trap (NET) formation. Neutrophil homeostasis is regulated through balancing granulopoiesis, the retention of produced neutrophils in the bone marrow, and their mobilization and attraction to peripheral tissues [35].

During bone marrow-located granulopoiesis, pluripotent hematopoietic stem cells, *via* an intermediate myeloid progenitor cell stage, constantly give rise to immature neutrophils, whose retention in or their mobilization from the bone marrow is dependent on a fine-tuned process involving growth factors as well as chemokine–chemokine receptor interactions [35]. Acute and chronic infections as well as chronic mental or physiological stress increase granulopoiesis and the mobilization of neutrophils from the bone marrow [35,36]. The main regulator of neutrophil granulopoiesis and survival, G-CSF, is produced by activated fibroblast and epithelial cells [24]. The cytokines interleukin (IL)-1β and IL-17A are the most potent inducers of G-CSF in dermal fibroblasts and keratinocytes [24]. Additionally, G-CSF is produced upon tissue injury and the recognition of microbial and damaged host cell components by tissue resident immune cells [24]. By upregulating transcription factors that direct the differentiation of myeloid progenitors towards the neutrophil lineage, G-CSF directly impacts the size of the neutrophil reservoir in the bone marrow [35]. Furthermore, G-CSF-mediated downregulation of the bone marrow-homing receptor CXCR4 expression on neutrophils and simultaneous upregulation of the neutrophil-attracting chemokine CXCL1 by endothelial cells provoke neutrophil mobilization and attraction into the blood [35]. 

The limitation of strengthened granulopoiesis during subsidence of the inflammation is mediated by a negative feedback loop based on the decreased availability of G-CSF-inducing stimuli (e.g., IL-1β/IL-1β-inducing microbial and damage cell components). Moreover, after phagocytosis of apoptotic neutrophils (efferocytosis), monocytes/macrophages and dendritic cells show a reduced IL-23 production [37,38]. As IL-23 plays a key role in the development and maintenance of specific lymphocyte subtypes (Th17/γδ17/Tc17) that produce the G-CSF inducer IL-17 [24,39], the apoptosis of neutrophils may limit G-CSF-dependent granulopoiesis in the resolution phase of an infection. Interestingly, the phagocytosis of apoptotic cells by monocytes/macrophages is strengthened by IL-10 [40], a cytokine whose expression is highly produced in lesioned HS skin [41].

## 3. Role of Neutrophilic Granulocytes in HS Lesions

In HS lesions, the limited epidermal upregulation of antibacterial proteins enables the propagation of the microbes in the skin [20,41,42]. The persistent presence of bacterial components supports the chronic inflammation through the stimulation of monocytes/macrophages/dendritic cells *via* their innate immune receptors (i.e., pattern recognition receptors, PRRs) [41,43]. Cytokines, secreted by these cells (e.g., IL-1β, TNF-α) promote the skin infiltration (induction of specific chemokines such as CXCL1, CXCL6, CXCL8 [43] as well as LCN2 [25]), survival and activity (induction of G-CSF [24,43]) of neutrophils.

The expression of G-CSF is strongly upregulated in HS skin lesions compared not only to healthy donor skin, but also to lesioned skin from other chronic, inflammatory skin diseases such as psoriasis and atopic dermatitis [24]. Even though quantification of systemic G-CSF level reveals only a trend of increase in HS patients, a correlation with disease severity expressed by the Sartorius score was found [24]. G-CSF strengthens the expression of several transmembrane receptors in neutrophils that contribute to the prolonged activation of these cells by components of bacteria and the mitochondria of disrupted host cells (e.g., formyl peptide receptor 1 (FPR1), FPR2 and free fatty acid receptor 2 (FFAR2)) [24]. Furthermore, G-CSF upregulates the expression of the decoy receptor TNFRSF10C/TRAIL-R3 and TNFRSF6B, which prevent the effect of TRAIL and TNFSF6, respectively, and protect cells from apoptosis [24]. The most downstream elements of the G-CSF pathway in HS include proteases (e.g., ADAM8, MMP8, MMP9, MMP25) [24]. Thus, extracellular matrix-damaging and -degrading proteins (e.g., MMPs, myeloperoxidase, neutrophil elastase, cathepsins), secreted by neutrophils are assumed to contribute to the substantial tissue destruction observed in HS [20,23]. By supporting the rupture of dilated hair follicles, MMPs might promote abscess and tunnel formation in the chronic stage of HS. G-CSF could further drive those processes as it was shown to provoke an upregulation of ADAM8 and MMP8 in toll-like receptor 4-activated neutrophils [24]. It should also be mentioned that TNF-α is also able to directly activate neutrophils to produce MMP8 and LCN2 [23,25]. LCN2 is a soluble multifunctional glycoprotein. It transports small hydrophobic molecules and is involved in the induction of inflammatory pain. LCN2 also acts as a chemoattractant for neutrophils, promotes adhesion and extravasation of these cells, and may therefore contribute to purulent inflammation in HS [20,44].

Like G-CSF itself, the expression of the known G-CSF inducers IL-1β and IL-17 is also strongly increased in lesioned HS skin [41,43]. These cytokines induce G-CSF in fibroblasts (IL-1β) and keratinocytes (IL-17A) [24,45]. Accordingly, cutaneous IL-1β and IL-17A expression levels clearly correlate with the expression of G-CSF in lesioned HS areas [24]. Additionally, IL-1β and IL-17A are major inducers of neutrophil-attracting chemokines in fibroblasts and keratinocytes, respectively [43,45].

## 4. IL-1/IL-17/G-CSF Axis as Potential Target of Phytotherapy in HS

The field of phytotherapy comprises several candidates for the modulation of the purulent destructive inflammation characteristic of HS. Targets may include the immune mediators that are involved in the immigration of neutrophils into the skin, the G-CSF inducers, G-CSF itself, or other cytokines activating neutrophils in the skin. Within the wide variety of secondary phytochemicals, polyphenols are substances with high anti-inflammatory potential. Mechanisms underlying their anti-inflammatory effects comprise the inhibition of proinflammatory cytokine production, the interference with cytokine-induced signal transduction pathways, as well as the modulation of lymphocyte lineage development [46].

**Figure 1:**
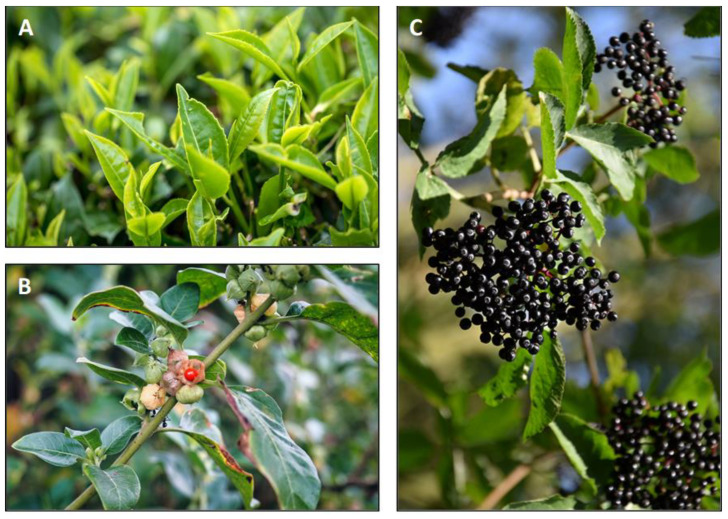
Botanical pictures of *Camellia sinensis* (**A**), *Withania somnifera* (**B**) and *Sambucus nigra* (**C**).

The transcriptional regulation of G-CSF is known to be mainly dependent on NF-κB signaling [47]. Accordingly, the signal transduction profile of the G-CSF inducers IL-1β and IL-17A includes activation of the NF-κB pathway [48]. IL-1β exerts its effects through binding to a heterodimeric receptor comprising IL-1R1/IL-1RAcp, while IL-17A mediates its biological effects through the heterodimeric IL-17RA/IL17RC receptor complex, which is shared by IL-17F as well as by IL-17A/IL-17F homo-/heterodimers [49,50]. Among phytochemicals, polyphenols derived from *Camellia sinensis* (epigallocatechine-3-gallate, EGCG), *Withania somnifera* (withaferin A) and red berries (cyanidin) are potential candidates for the therapeutic modulation of the IL-1β/IL-17A/G-CSF axis (Figure 1).

The tea plant (*Camellia sinensis*) is found in tropical and subtropical areas with a history in its agricultural use for tea preparation that spans more than 1500 years. The main polyphenolic constituents of *Camellia sinensis* are catechins (flavan-3-ols) and their derivatives. Among those, epigallocatechine-3-gallate (EGCG) as well as a whole polyphenol mixture prepared as green tea extract are the most studied phytochemicals of *Camellia sinensis*. It is assumed that EGCG is a natural ligand of the 67 kDa laminin receptor (67LR) [51]. The winter cherry (*Withania somnifera*) is a common plant predominantly found in Mediterranean regions, with a long history of its use in ayurvedic medicine. Among the withanolides, the secondary phytochemicals present in the roots of *Withania somnifera*, withaferin A (steroidal lactone) are the most studied one. Cyanidin as well as its derivatives (e.g., cyanidin-3-glucoside) belong to the group of anthocyanidins and are commonly found in red berries and some fruits, e.g., black elderberries (*Sambucus nigra*), chokeberries (*Aronia melanocarpa*), or blackberries (*Rubus fruticosus*). The authors note that phytochemical substances mediate differential effects on primary and tumor cells [52,53]. This review therefore primarily focuses on the scientific work based on primary cells. 

## 5. IL-1β Pathway Modulators

**Figure 2:**
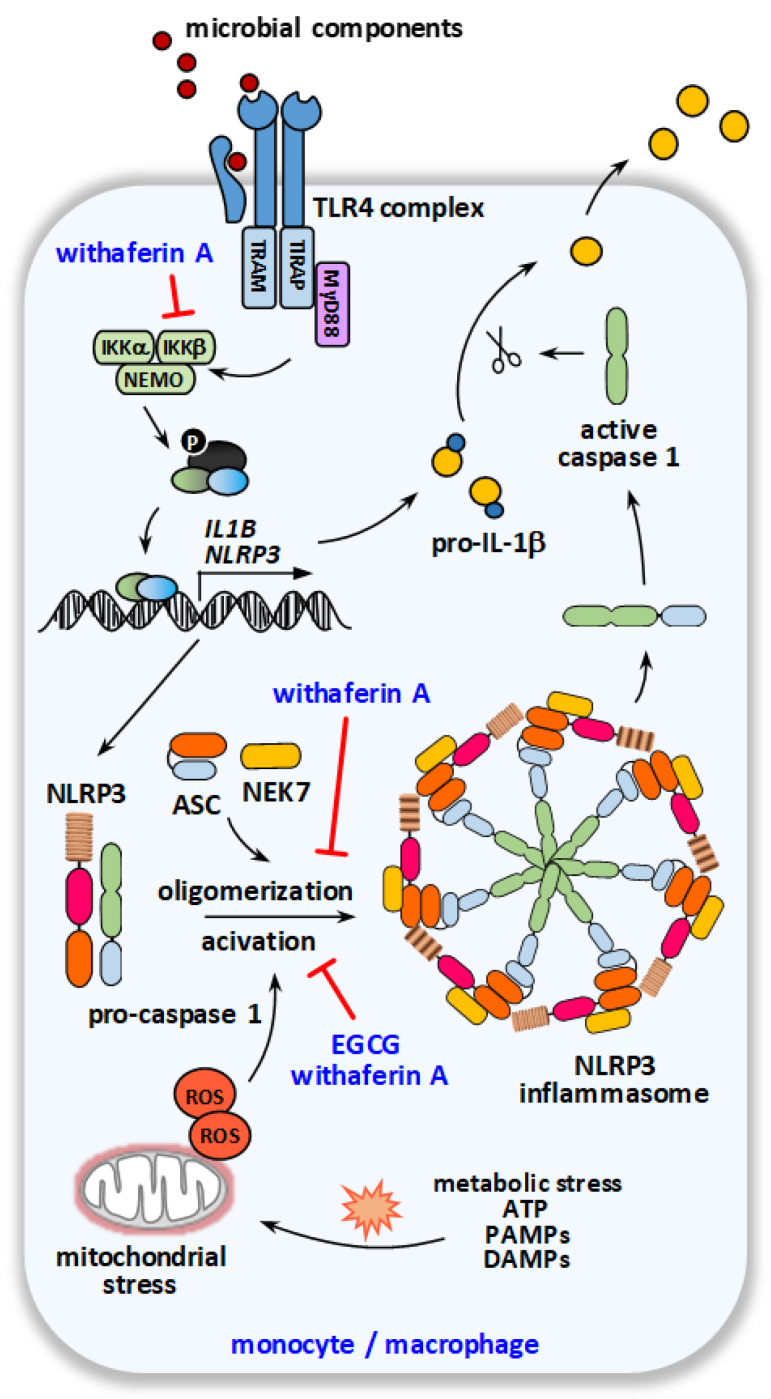
**Schematic overview on the molecular targets of EGCG and withaferin A within the IL-1β pathway**. Recognition of microbial components by pattern recognition receptors (priming) on monocytes/macrophages leads to upregulation of NLRP3 inflammasome components and their subsequent oligomerization resulting in NLRP3 inflammasome assembly. As a second signal, e.g., metabolic stress and mitochondrial dysfunction lead to activation of the NLRP3 inflammasome, autoactivation of pro-caspase-1, and thereby enables caspase-1-mediated cleavage of pro-IL-1β to IL-1β as its active form.

A potential candidate for the modulation of the IL-1β pathway is the polyphenol EGCG, derived from the tea plant (*Camellia sinensis*), as well as withaferin A from *Withania somnifera* (Figure 2).

As IL-1β is secreted in an inactive form (pro-IL-1β), it has to be activated by the inflammasome to confer its biological activity, a mechanism that enables the fine-tuned regulation of immune activation to prevent excessive inflammation and inflammatory cell death (pyroptosis) [54]. Thereby, the conversion of pro-IL-1β to active IL-1β is initiated by the oligomerization and activation of the NLRP3 (NLR family pyrin domain containing 3) inflammasome, a multi-protein complex consisting of NLRP3, caspase-1 and the adaptor accessory protein ASC (apoptosis-associated speck-like protein containing a CARD) and NEK7 (NIMA-related kinase 7), and subsequent caspase-1 mediated cleavage. Components of the NLRP3 inflammasome have been shown to be upregulated in HS lesions [43,55].

In murine macrophages, EGCG was described to prevent IL-1β induction, possibly by inhibiting NLRP3 activation [56]. A potent NLRP3 inhibitory property of EGCG and the subsequent prevention of caspase-1 activation and IL-1β secretion was also found by Zhang et al. [57]. In line with these data, EGCG limited ex vivo lymphocyte-derived, as well as systemic, IL-1β levels using different in vivo models [58,59]. Moreover, the PPR (AIM2)-dependent IL-1β secretion of keratinocytes in response to a dsDNA analog was suppressed by EGCG in vitro [60].

In the course of scientific investigations on the therapeutic effects and mode of action of withaferin A, this phytochemical has been shown to be an effective inhibitor of NLRP3 activation. In fact, the *Helicobacter pylori*-induced upregulation of NLRP3 and IL-1β in bone marrow-derived dendritic cells was limited by withaferin A treatment [61]. Xia et al. also reported a dose-dependent decrease of LPS-induced NLRP3 and IL-1β mRNA expression in primary murine macrophages, whereby this effect was diminished in NLRP3^-/-^ macrophages [62]. In THP1 macrophages, NLRP3 activation was counteracted by withaferin A, associated with a dose-dependent suppression of the bacterial component-induced upregulation of IL-1β at the mRNA and protein level [63]. The authors suggested an impaired co-localization of NLRP3 inflammasome elements to be a possible mechanism underlying this withaferin A effect [63]. Withaferin A also prevented NLRP3 activator-induced cleavage of inactive pro-caspase-1 and pro-IL-1β in a dose-dependent manner [61]. Furthermore, in a chronic pancreatitis model, withaferin A was observed to prevent cerulein-induced pancreatic NLRP3 and ASC mRNA upregulation [64]. The inhibitory effect of withaferin A on NLRP3 activation and IL-1β secretion was also confirmed using an ovalbumin-induced airway inflammation model [65]. Moreover, the suppressing effect of withaferin A on hepatic NLRP3 inflammasome activation and systemic IL-1β induction was observed using an in vivo hepatitis model [62].

## 6. IL-17 Pathway Modulators

**Figure 3:**
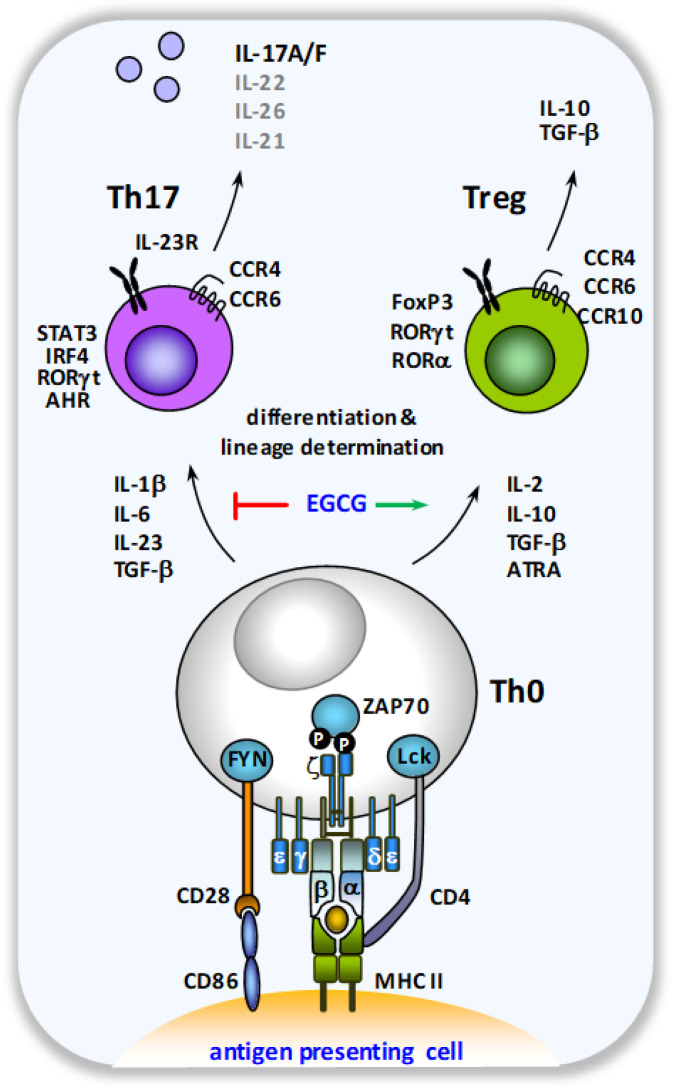
**Schematic overview of the modulation of Th cell lineage development by EGCG**. When activated by antigen-presenting cells, naïve CD4^+^ T cells can differentiate into various effector cell subtypes, depending on the local cytokine milieu. In the presence of an inflammatory cytokine milieu, dominated by the presence of IL-1β, IL-6, IL-23 and TGF-β, Th17 lineage determination is favored with the concomitant production of the Th17 cell signature cytokine IL-17A.

**Figure 4:**
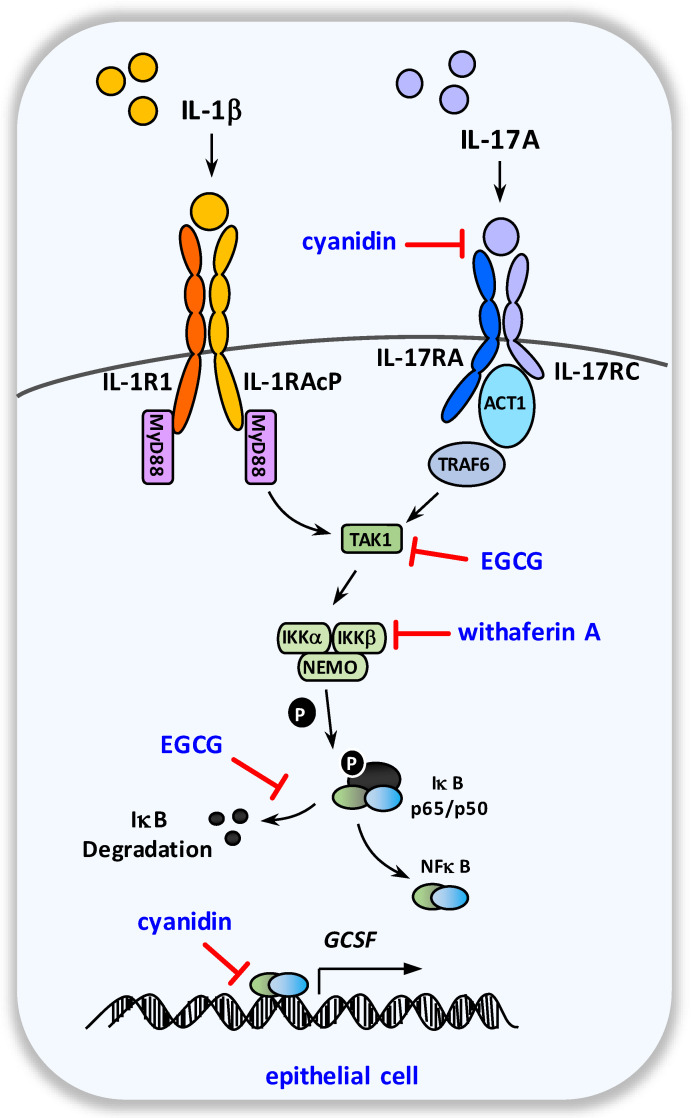
**Schematic overview of the molecular targets of EGCG, withaferin A, and cyanidin within the G-CSF pathway**. After binding to and signaling through their specific heterodimeric receptors, the G-CSF-inducers IL-1β and IL-17A provoke NF-κB activation and the subsequent upregulation of G-CSF expression in epithelial cells.

Within the field of phytotherapeuticals, potential IL-17A pathway modulators include the polyphenols EGCG from *Camellia sinensis* and cyanidin from red berries (Figure 3 and Figure 4). Cyanidin was first identified as a potent inhibitor of IL-17A based on a bioinformatic approach, followed by in vitro and in vivo evaluations [66]. Molecular interaction analyses revealed that cyanidin binds to the IL-17A-binding site of its IL-17RA receptor chain [66]. Cyanidin prevented IL-17A-induced chemokine production associated with reduced phosphorylation of IκBα in vitro. Furthermore, IL-17A-mediated skin hyperplasia was attenuated by cyanidin in vivo [66]. Using an in vivo asthma model, cyanidin was also shown to prevent ovalbumin-specific Th17- but not Th2-mediated inflammation and neutrophilia [66]. Additionally, in an experimental autoimmune encephalomyelitis model, cyanidin treatment attenuated the disease activity score in myelin oligodendrocyte glycoprotein-specific Th17, but not Th1 adopted mice [66].

In different preclinical models of neutrophilic airway disease, asthma and COPD, cyanidin-3-glucoside (a cyanidin derivative with similar IL-17A/IL-17R blocking capacity) substantially suppressed neutrophilic inflammation and restored dexamethasone sensitivity [67]. In monocytes and synoviocytes obtained from arthritis rats, the IL-17A-mediated migration and target gene expression was alleviated by cyanidin [68,69]. In a further study using this model, respective mice also showed a normalized IL-17 and IL-10 blood level indicating an influence on the Th17/regulatory T cell balance [70]. However, further studies are needed to validate these findings. 

Decreased Th17 cell numbers in favor of enhanced proportions of regulatory T cells, accompanied by reduced lymphocyte IL-17A secretion and disease severity reduction, were also detected after EGCG treatment in murine models of autoimmune encephalomyelitis, arthritis, colitis, and obesity [59,71,72,73,74]. In line with these data, EGCG impeded Th17 lineage development by the modulation of murine-naïve T helper cell differentiation [74,75]. Furthermore, Th17 polarization of murine splenocytes was reduced by EGCG treatment [74]. The underlying mode of action is suggested to involve the EGCG-mediated suppression of mTOR activation and the subsequent abrogation of hypoxia-inducible factor 1 (HIF-1a)-dependent RORγt activation, a molecular checkpoint for Th17/Treg lineage development described earlier [73,74,76,77].

EGCG may also directly interfere with IL-17A target gene expression. The suggested mechanism is the modulation of respective signal transduction pathways. In fact, by inhibiting the IL-17-dependent activation of mitogen-activated protein kinases (p38 and ERK), EGCG decreased IL-17A target gene expression in fibroblasts [78]. However, these data have to be validated in further studies.

## 7. TNF-α/NF-κB Pathway Modulators

Phytochemicals targeting the NF-κB pathway include the polyphenols EGCG, withaferin A and cyanidin (Figure 4). Inhibition of NF-κB pathway activation by EGCG was shown in various in vitro cell culture models [79,80,81,82]. Analysis on the underlying mechanisms revealed that EGCG targets specific key points within the NF-κB signaling pathway. In Fact, EGCG was reported to prevent the degradation of IRAK and thereby inhibited NF-κB activation in respiratory epithelial cells in vitro [82]. In contrast, Singh et al. did not find prevention of IRAK degradation by EGCG in rheumatoid synovial fibroblasts cells but instead found inhibition of IRAK activity [81]. Furthermore, using synovial fibroblasts, EGCG was found to represent a potent inhibitor of TAK1 kinase, a signaling molecule upstream of NF-κB, activated by preventing its phosphorylation at Thr^(184/187)^ [80,81]. Accordingly, EGCG effectively prevented IL-1β-induced NF-κB p65 nuclear translocation in the study by Fechtner et al. [80]. Beside its ability to interfere with canonical NF-κB (IKKα/β) signaling, in silico analysis further revealed that EGCG might also block the non-canonical NF-κB pathway molecule NF-κB induced kinase (NIK) [83].

Withaferin A was also reported to possess NF-κB-inhibiting activity in various cell types [61,64,84,85,86,87]. Kaileh et al. found that this phytochemical is able to prevent IκBa degradation by inhibiting IKKβ activity in vitro [84]. Subsequently, the work of Heynink et al. further elucidated the mode of action of withaferin A-mediated IKKβ inactivation [88]. This study revealed that withaferin A specifically targets the Cys^179^ residue of IKKβ, thus inhibiting its catalytic activity [88]. In line with its described NF-κB pathway-modulating property, withaferin A suppressed the spontaneous and bacterial component-induced IL-1β production in immune cells obtained from rheumatoid arthritis patients [87]. Moreover, paw swelling was effectively prevented by withaferin A in vivo*,* using a zymosan-induced paw inflammation model [84]. In a chronic pancreatitis model, withaferin A suppressed pancreatic neutrophil infiltration and the nuclear translocation of the NF-κB p65 subunit in acinar cells [63]. The therapeutic suitability of *Withania somnifera* as a potential NF-κB modulator has also been of clinical interest. A respective randomized phase II clinical trial is currently ongoing (clinicalTrials.gov Identifier: NCT05031351; accessed on 1 August 2022).

Cyanidin represents a further phytochemical modulator of the NF-κB pathway. In fact, cyanidin was found to be a very potent small molecule activator of sirtuin-6, a histone H3 deacetylase [89]. Sirtuin-6 itself was shown to interact with NF-κB p65 subunit (RelA) and to deacetylate the histone H3 lysine 9 position (H3K9) at NF-κB target gene promoters, thereby impeding transcription of NF-κB dependent genes [90,91]. Furthermore, cyanidin also inhibited NF-κB signaling by the suppression of IκBα degradation and NF-kB translocation [92,93,94,95]. Accordingly, the attenuation of NF-κB mediated inflammation by cyanidin and cyanidin-3-glucoside in vivo was observed [92,93,94,96].

## 8. Conclusions

Considering the massive burden HS patients have to carry, including the substantial negative impact on the patients’ personal life, professional life and life expectancy, HS is a huge clinical challenge for dermatology [30].

Complementary therapy options are already an established part in dermatology. However, phytochemicals are not taken into account as complementary approaches so far, though their therapeutic potential could offer an important contribution to an integrated HS management. There are substantial data suggesting the clinical use of phytochemicals derived from *Withania somnifera*, *Camellia sinensis* as well as cyanidin (from red berries) for the modulation of the IL-1/IL-17/G-CSF axis in HS. Overall, data from respective preclinical studies on these substances reveal several interesting findings and contribute to our understanding of underlying mechanisms of their action.

Before starting an integrative HS management, a detailed and careful analysis of potential drug interactions, also considering the concomitant medications of the patient, should be made. In general, phytomedical therapy should only be performed under strict medical supervision and monitoring, considering the individual clinical situation of the patients and the evaluation of current data.

### Safety and Drug Interactions of Phytomedicals for Integrated HS Therapy

In general, the clinical use of phytochemicals derived from *Withania somnifera* was found to be tolerable and safe, indicated by pharmacokinetics and safety data obtained from clinical trials [97,98,99,100,101,102,103]. The clinical use of *Camellia sinensis*-derived phytochemicals was extensively studied in clinical trials and respective pharmacokinetic and safety data are available [104]. Based on these data, the upper safe dosage limits of 338 mg EGCG (in form of an extract) or 704 mg EGCG (consumed as beverage) for the clinical use of these substances are recommended [104]. For cyanidin and its derivatives, no safety concerns are indicated from clinical studies evaluating pharmacokinetics, tolerability and safety so far, whereby these data have to be substantiated in further studies [105,106,107,108].

Among the phytomedical drugs evaluated here, most reservations were raised about the clinical use of catechins derived from *Camellia sinensis*. This relates partly to their interaction with cytochrome P450-metabolizing/detoxifying enzymes, having the potential to modify the efficacy of cytochrome P450 metabolization of concomitantly given drugs [109,110,111]. Additionally, EGCG and other *Camellia sinensis* catechins are natural inhibitors as well as substrates of the enzyme catechol-o-methyltransferase (COMT), which is required for the detoxification and metabolization of xenobiotics (i.g. levodopa, isoprenaline, benserazide), catecholamines and catechol estrogens, promoting their excretion and preventing oxidative stress or carcinogenesis [112]. As catechol-containing flavonoids from *Camellia sinensis* might compete in vivo with those substrates for COMT-mediated metabolization, possible interactions with current medication should be considered, especially for those patients carrying the low-activity COMT genotype [112]. Where appropriate, assessment of the COMPT genotype is therefore suggested before starting green tea extract or ECGC treatment. Furthermore, as liver toxicity has been proposed as a rare adverse reaction, treatment of patients showing hepatic dysfunction with *Camellia sinsensis* catechins should be avoided as safety precaution.

Special caution should also be made for diabetic patients before staring *Camellia sinensis*-derived catechins. In fact, these substances should be administered only postprandially, as the achievement of protective effects on glucose metabolism is probably limited to their gastrointestinal effects [113]. In fact, a negative impact on glucose metabolism was reported when EGCG was applied preprandially, but whether this might be a result of inhibited tissue glucose uptake is still under debate [113,114]. However, data on the preprandial effects of tea catechins need to be confirmed in larger studies. Moreover, for EGCG, a histone deacetylase-inhibitory potential has been proposed, but remains to be proven in vivo [115]. 

For *Withania somnifera* root extract it is not finally clarified as to whether there is an interaction with cytochrome P450 enzymes or choline esterases [116,117,118,119]. However, precaution regarding respective possible adverse effects or drug interactions with concomitant medication including modulation of drug efficacy is indicated.

Furthermore, it has to be noted that an improvement of thyroid function after therapy with *Withania somnifera*-derived root extract has been observed in subclinical hypothyroid participants, including a significant reduction in thyroid-stimulating hormone levels (TSH) and a concomitant increase of triiodothyronine (T3) and thyroxine (T4) levels [102]. A constant monitoring of thyroid parameters is thereby recommended during the *Withania somnifera*-based treatment of hypothyroid patients with concomitant L-thyroxin, as well as of hyperthyroid patients. On the other hand, subclinical hypothyroid patients might benefit from the described effect on thyroid function. Of note, using an obesity mouse model, withaferin A was shown to represent a potent leptin sensitizer [120]. Therefore, changes in lipid/glucose parameters should be considered as possible effects of *Withania somnifera* preparations and carefully monitored under treatment.

In summary, the evaluated data indicate that phytomedical drugs derived from *Withania somnifera*, *Camellia sinensis* and red berries may represent potential modulators of cytokines that play a decisive role in promoting the granulocyte-associated pathogenetic destructive inflammation of HS skin. Respective clinical trials are now needed to evaluate the potential and suitability of these substances as an element of an integrated therapeutic strategy for HS. Based on the clinical trial by Czank et al. and the so far reported safety profile, especially cyanidin and its derivatives might be an appropriate candidate for setting up a first clinical pilot trial in this regard [105].

## Data Availability

Not applicable.
